# Hand-held device to remove a single-rod subdermal contraceptive implant: Results of early trials in Sweden ^☆☆^

**DOI:** 10.1016/j.contraception.2020.07.005

**Published:** 2020-07-18

**Authors:** Karin Emtell Iwarsson, Ami Sakaria, Marte Bratlie, David Hubacher, Kristina Gemzell-Danielsson

**Affiliations:** a Department of Women’s and Children’s Health, Karolinska Institutet, WHO-centre, QB:84, Karolinska vägen 37 A, Karolinska University Hospital, SE-171 76 Stockholm, Sweden; b RemovAid AS, Forskningsparken, Gaustadalleen 21, 0349 Oslo, Norway; c Department of Product Development and Introduction, FHI 360, 359 Blackwell Street, Durham, NC 27701, USA

**Keywords:** LARC, Subdermal contraceptive implant, Device removal, Implant removal service

## Abstract

**Objective:**

Long-acting reversible contraceptives (LARCs) such as subdermal implants and intrauterine devices are promoted and increasingly used worldwide. Hence, in the light of this we also need to ensure easy access to the reversibility, i.e. emphasise the R in LARC. Our overall aim is to develop a device to facilitate implant removals. We evaluated the safety and performance of the two initial field prototypes where the main outcome was percentage of successful fixations and secondary outcomes were percentage of successful removals without the use of additional tools, duration of the procedure, satisfaction and adverse events.

**Study design:**

We performed a feasibility study including 41 subjects.

**Results:**

We estimated a fixation rate of 35/41 (85%) and an overall removal rate of 24/41 (59%). Further, we measured that the median time for removals was 80 s and that subjects and operators were satisfied with the procedure. We recorded adverse events such as bruising and superficial abrasions.

**Conclusions:**

The device demonstrated a successful fixation rate, however, the removal rate will need to be further improved.

**Implications:**

This feasibility study shows that the device has potential and further research is needed to modify and optimize the device.

## 1. Introduction

Long-acting reversible contraceptives (LARCs) such as subdermal implants and intrauterine devices are promoted [1] and increasingly used worldwide [2]. To ensure optimal user functionality, women need easy access to removals.

An implant removal procedure is generally considered to be minor surgery [3]. The standard removal technique for palpable single-rod implants is typically performed with local anaesthesia in aseptic conditions, using scalpel and forceps to incise the skin and extract the implant, respectively [4-6]. Removals require trained health-care providers, adequate facilities and sterile instruments [7]. Lack of skilled experienced operators, geographical isolation or other reasons can prevent timely removal [3]. Implants are typically inserted using a dedicated insertion trocar, however, no device exists to remove them.

**Fig. 1.**
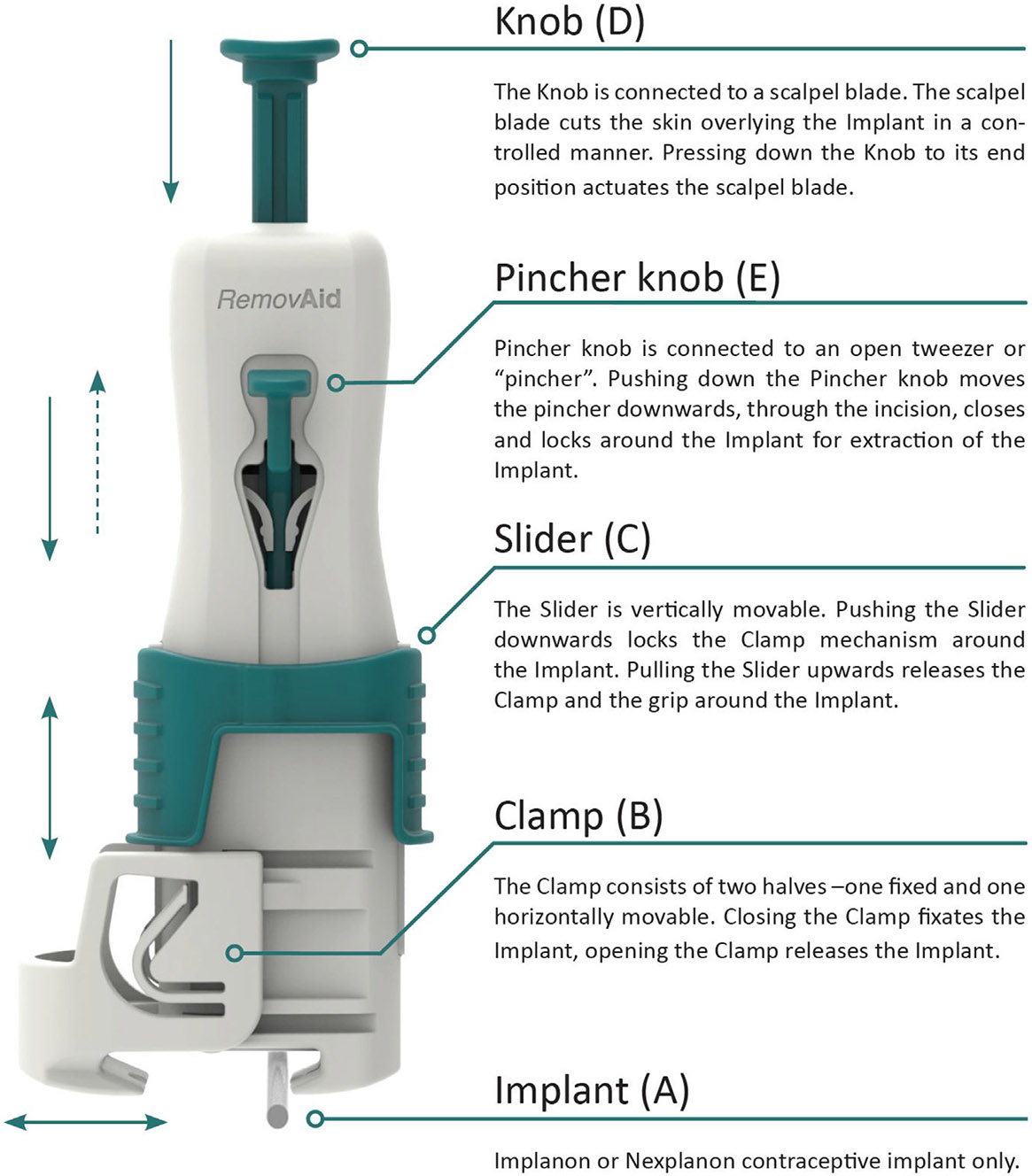
Overview of the RemovAid^™^ device.

RemovAid AS of Norway developed a hand-held device for removal of a single-rod implant (RemovAid^™^). The RemovAid device is a single-use, sterile, hand-held device intended to extract single-rod contraceptive implants i.e. Implanon/Nexplanon (Fig. 1). The device is 13 cm in length and has a built-in scalpel blade that incises the skin transversely over the implant at the midpoint. The device fixates around the skin and implant within its clamp, and the scalpel center does not penetrate beyond the clamp – ensuring superficial incisions [8].

We assessed the following main and secondary outcomes: percentage of successful fixations (main outcome), removals without the use of additional tools, duration of the procedure (from time of incision to time of extraction). Further, we assessed inter operator variability, product functionality evaluated by the operators and satisfaction of the removal procedure rated by the subjects. Finally, we collected all reports of adverse events (AEs) and serious adverse events (SAEs).

## 2. Material and methods

We conducted a feasibility study, according to the National Institute for Health Research’s definition of feasibility studies, out of two single-arm pilot trials. We wanted to estimate important parameters to design further randomized controlled trials.

We included subjects if they (i) were 18 years or older, (ii) had a palpable one-rod subdermal implant, (iii) were willing to provide follow-up information. We excluded subjects with these characteristics: a known allergy to local anaesthetics or disinfectant, an active skin lesion over the implant’s location, or any disorder or medication potentially affecting the coagulation status, the wound-healing process or increasing the risk of infection.

We conducted the study at the WHO Collaborating Centre in Stockholm, Sweden. We recruited subjects using advertisements and from outpatient clinics. We enrolled subjects at the centre if they fulfilled the inclusion criteria and had no exclusion criteria.

After participants signed the informed consent, the operator disinfected the skin with Chlorhexidine, applied anaesthesia (lidocaine) either as an injection or with local topical patches, fixated and removed the implant with the device, and closed the incision with wound tape and bandage. For those where the implants could not be removed with the device, the operator removed the implant with standard removal equipment, preferably through the incision created by the RemovAid device.

We performed a follow-up at 7 days (±1 day) for inspection of the removal site, and reports of eventual adverse events (AEs).

Two nurse-midwives and two gynaecologists, proficient in standard removal techniques, performed all the procedures; they received instruction on the device and trained on arm models before study start.

We defined feasibility as the successful fixation rate (main outcome), where our pre-specified desired fixation rate would be 80% or more. Further, we defined feasibility as the successful removal rate (secondary outcome), where our pre-determined desired removal rate would be 90% or more of all implants that were successfully fixated.

Operators scored their overall impression of the device, i.e. the functionality, and subjects scored their overall impression of the procedure with the device, i.e. their satisfaction. Both measures used a five-point scale (5 = Excellent, 4 = Very good, 3 = Good, 2 = Somewhat dissatisfied, 1 = Very poor/dissatisfied).

We generated descriptive statistics including medians, range and other measures of dispersion. We used IBM SPSS^®^ Statistics for Mac, version 25 for analyses.

## 3. Results

**Fig. 2.**
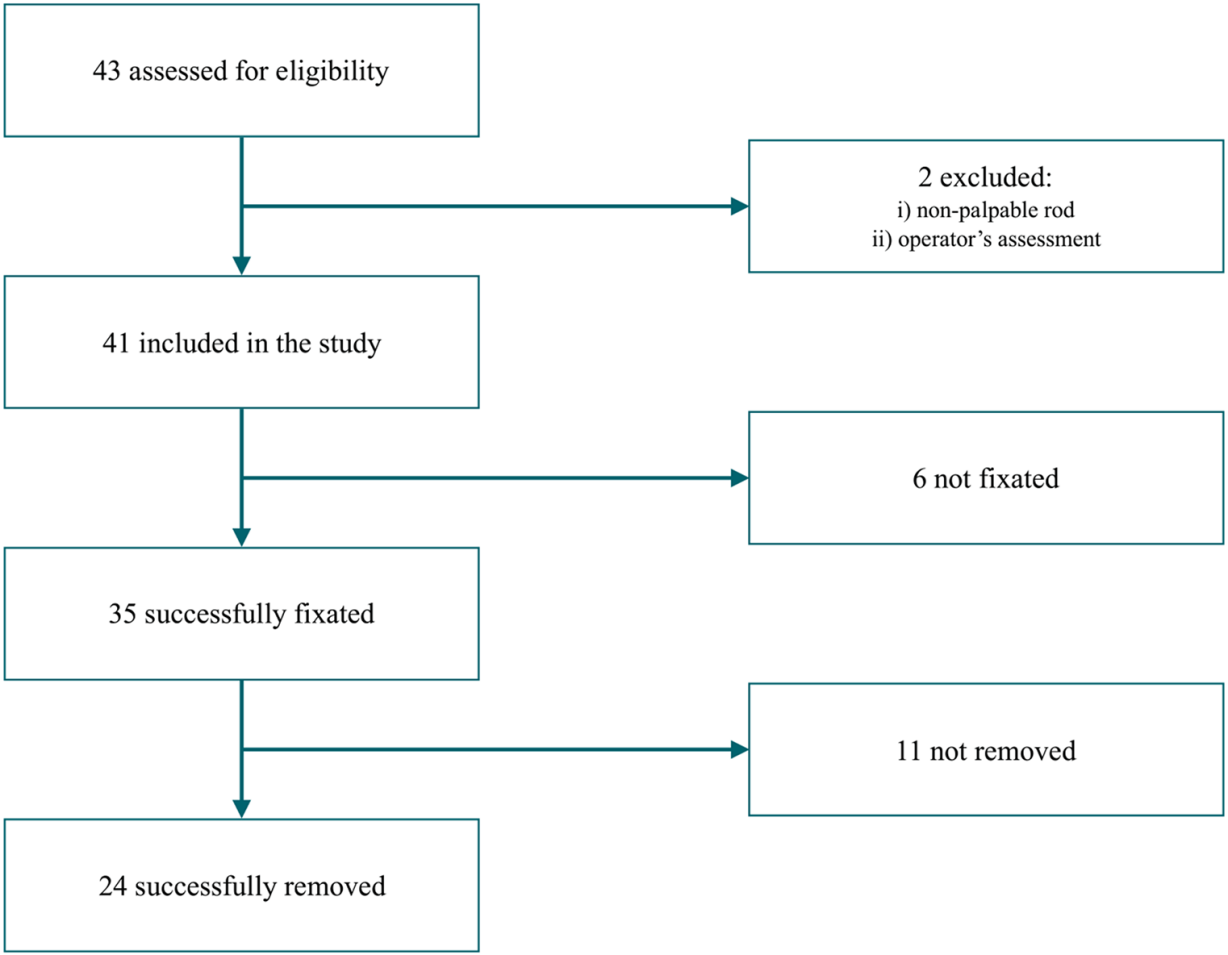
Disposition of subjects.

We included 41 subjects (Fig. 2) from January 2017 to June 2018.

Implant users had a median age of 23 (range 18–35) and a median body mass index (BMI) of 25 (range 18–33). The median time since insertion was 35 (range 4–84) months. The most commonly used anaesthesia 35/41 (85%) was patches. The majority of the rods (26/41, 63%) were assessed as being placed superficially or very superficially.

Of the total cohort, we can conclude that 35/41 (85%) rods were successfully fixated and 24/41 (59%) were successfully removed. Further, we found that 24/35 (69%) of the fixated implants were successfully removed using the device. The operator with the most removal attempts, successfully removed 7/14 (50%) of the rods and the one with the least removed 4/8 (50%). The most successful operator removed 7/10 (70%) and the least successful operator removed 7/14 and 4/8 (50%).

Our results show that the total cohort’s median removal time was 80 s (range 34–230 s). The median removal time for the slowest and the fastest operator was 135 s (range 54–230 s) and 67 s (range 34–113 s) respectively.

The subject’s median satisfaction of the removal was 5, where 92% rated 3 or above. The operators’ median functionality of the device was 4, where 69% rated 3 or above. Both subjects and operators have reported the ratings for the total cohort i.e. regardless of the removal success.

We found that the most frequently occurring AE was a superficial abrasion (a small puncture a few mm from the main incision site), which occurred in 12 out of 30 reported events (40%). Bruising occurred in 30%. We had no reported SAEs.

## 4. Discussion

Our aim of the study was to evaluate the safety- and performance of RemovAid^™^. We found no safety concerns, the implant fixation rate was 85% and the removal rate 59%. Further, 69% of the fixated implants were removed.

We did not reach the pre-determined desired rate (90% or more) for removed implants however, we reached the pre-determined successful fixation rate (80% or more). This indicates that the device will need further optimization before a larger clinical trial can be performed.

The operator who performed the most procedures and the ones who performed the least had equal removal success rate (50%), which indicate that a higher removal success rate did not depend on experience.

Our median duration for a successful removal procedure (80 s) was similar to standard removals (1 min to <4 min) [4,9]. Furthermore, this study showed the inter-variability of the operators’ median removal time was as short (approximately 1 min).

Both operators and subjects gave high ratings regarding their general impression of the removal session and the device, indicating that the device is acceptable both to subjects and operators.

Bruising is a commonly reported AE associated with the standard removal procedure [6]. The frequency of bruising detected in this study is similar to the standard removal procedure. Also, some subjects had implants reinserted in the same removal site which can increase incidence of bruising [6].

No dedicated product for implant removal exists. A fully optimized device with higher efficacy, could be used in many different settings and have numerous advantages. For example, in sub-Saharan Africa where implant insertions have increased dramatically since 2013 [10], concerns have been raised about lack of access to removal due to shortages of trained/competent providers, particularly in rural areas [11]. A recent study in Ghana found that only 61% of implant users were successful in having their implant removed on their first attempt at seeking services [12]. An easy-to-use device can enable lower-level providers to deliver services, thereby reducing barriers to timely removal. In addition, an all-in-one device can have these advantages: obviate need to sterilize an assortment of standard removal tools, eliminate scalpel injuries and cross-contamination risks, eliminate variability in handling a scalpel that could lead to serious patient injury. Some of these advantages are relevant to even resource-rich countries.

The work continues to improve and test RemovAid^™^: a third pilot study in Sweden using a modified device and a randomized controlled trial is taking place in Uganda.

## Supplementary Material

Supplementary
